# *GSTM1* and *GSTT1* Genetic Polymorphisms and Breast Cancer Risk in Selected Filipino Cases

**DOI:** 10.31557/APJCP.2019.20.2.529

**Published:** 2019

**Authors:** Noel Angelo Kalacas, Julius Adrie Garcia, Teresa Sy Ortin, Alfredo Valdez Jr, Allan Fellizar, Maria Cristina Ramos, Pia Marie Albano

**Affiliations:** 1 *The Graduate School,*; 2 *Research Center for the Natural and Applied Sciences,*; 5 *College of Science, University of Santo Tomas,*; 3 *University of Santo Tomas Hospital, Benavides Cancer Institute, Manila,*; 4 *Mariano Marcos Memorial Hospital and Medical Center, Batac, Ilocos Norte, Philippines.*

**Keywords:** Breast cancer, *GSTM1*- *GSTT1*, genetic polymorphism

## Abstract

**Background:**

The association of genetic polymorphisms with cancer development has been shown to be race- and tumor site-specific. Thus, this study aimed to determine whether polymorphisms in the *GSTM1* and *GSTT1* genes are associated with breast cancer among selected Filipinos.

**Methods:**

A total of 136 histologically confirmed breast cancer cases were age- and sex-matched with 136 clinically healthy controls. Genomic DNA extracted from blood samples of participants were screened for *GSTM1* and *GSTT1* genetic polymorphisms by multiplex PCR.

**Results:**

The frequency of null genotypes among the cases (*GSTM1*: n=78; 57.4%; *GSTT1*: n=61; 44.9%) was not significantly different (p>0.05) from the controls (*GSTM1*: n=93; 68.4%; *GSTT1*: n=59; 43.4%). It was also demonstrated that risk for breast cancer was increased in passive smokers carrying the *GSTM1* null (OR=2.56; 95% CI=1.38-4.75) or *GSTT1* positive (OR=2.00; 95% CI=1.05-3.83) genotypes. Moreover, risk was decreased in alcohol users carrying the *GSTT1* null (OR=0.39; 95% CI=0.16-0.97) genotype

**. Conclusion:**

This study suggests that variants of *GSTM1* and *GSTT1 *may not be risk factors for breast cancer development among Filipinos. However, the risk may be increased when these genotypes were combined with lifestyle or environmental factors.

## Introduction

Polymorphism is a genetic variation resulting in the occurrence of several different forms or types of individuals among the members of a single species. One of the most widely known types of genetic polymorphisms among people is the single nucleotide polymorphism (SNP), which involves a variation at a single position in a DNA sequence and occurs in more than one percent of the general population. SNPs have been found to help predict an individual’s response to certain drugs, susceptibility to environmental factors, and risk of developing diseases including cancer (Erichsen and Chanock, 2004). 

Genes that encode xenobiotic metabolizing enzymes (XMEs) are being reviewed continuously due to their essential functions in the detoxification of toxic compounds implicated in carcinogenesis (López-Cimam et al., 2012). The reactions catalyzed by these enzymes are classified into phase I and phase II reactions. Phase I system utilizes enzymes that initiate the detoxification process by transforming lipid-soluble substances into water-soluble compounds through the introduction of polar and reactive groups into the incoming substrate (Guengerich, 2001). Meanwhile, phase II system is responsible for conjugating the compounds modified and activated in phase I to specific substrates, yielding more polar and less toxic groups (Ford et al., 2000).

Among the most widely reviewed genes encoding the phase II detoxifying enzymes are glutathione S-transferase μ1 (*GSTM1*) and glutathione S-transferase θ1 (*GSTT1*). These genes are implicated in the conjugation of numerous carcinogenic compounds to excretable hydrophilic compounds (Hishida et al., 2005). However, null alleles of these genes corresponding to the deletion of whole protein-coding region have been identified to exist and are common across major human populations (Agúndez and Ladero, 2008). Individuals who completely lack *GSTM1* and *GSTT1* enzyme activity due to the inherited homozygous loss of these genes are more likely to have a higher risk of acquiring cancer (Ford et al., 2000).

Meta-analyses have shown the association of polymorphisms in *GSTM1* and *GSTT1* with increased breast cancer risk in Asians, most notably in Chinese populations (Tang et al., 2015; Xiao et al., 2015; Song et al., 2016). Numerous studies have also reported the possible association of deletions in *GSTM1* and *GSTT1* with the risk of developing other cancer types, including colorectal cancer in Caucasians (Economopoulos and Sergentanis, 2010), lung cancer in African-Americans, Japanese, Swedes, and South Indians (Ford et al., 2000; Sunaga et al., 2002; Alexandrie et al., 2004; Leelakumari et al., 2005), acute lymphoblastic leukemia in Thai (Pakakasama et al., 2005), and myeloid leukemia in non-Hispanic Caucasians (Davies et al., 2000). 

On the contrary, several studies have reported the absence of association of *GSTM1* and *GSTT1* polymorphisms with malignancies of the breast, colon, lung, prostate, and chronic myelogenous leukemia among Icelanders (Gudmundsdottir et al., 2001), Chinese (Economopoulos and Sergentanis, 2010), Greeks (Dialyna et al., 2003), and Japanese (Nakazato et al., 2003; Hishida et al., 2005), respectively. Acute lymphoblastic leukemia has been associated with *GSTM1* null but not *GSTT1* null genotypes in Filipino children (Rimando et al., 2008).

Those studies indicate that the association of polymorphisms in the *GSTM1* and *GSTT1 *genes with cancer is tumor site- and race-dependent. Hence, this study analyzed the association of polymorphisms in the *GSTM1* and *GSTT1* genes in a set of Filipino breast cancer cases and matched clinically healthy controls. The results of the molecular analyses were also associated with other risk factors for breast cancer.

## Materials and Methods

*Study Subjects*

Prior to the execution of this study, ethical clearances were secured from the Institutional Review Board (IRB) of the University of Santo Tomas Hospital (USTH) in Manila (Protocol Reference No. IRB-MD-09-2015-133) and the Mariano Marcos Memorial Hospital and Medical Center (MMMH-MC) in Ilocos Norte (RERC Protocol Nos. MMMH-RERC-15-005 and MMMH-RERC-15-006), Philippines. A total of 136 Filipino patients with histologically confirmed breast cancer, seen at the USTH and MMMH-MC from December 2015 to July 2016 were recruited for this study. The cancer cases were age- (±2 years) and sex-matched with clinically healthy controls who were not suspected to have any type of malignancy. All participants gave their written informed consent and were asked to accomplish through interview by a member of the research team a standardized questionnaire inquiring on their risk factors. Clinical data of the cases were retrieved from medical records and histopathologic reports.

*Genotyping of GSTM1 and GSTT1 Genetic Polymorphisms*

Genomic DNA was isolated from peripheral blood leukocytes of cancer cases and controls using ReliaPrep™ Blood gDNA Miniprep System (Promega, Madison, USA), following the manufacturer’s protocol. Genotyping for *GSTM1* and *GSTT1* was carried out by multiplex polymerase chain reaction (PCR) as described (Rimando et al., 2008) but with minor modifications. The primer sequences used for the detection of *GSTM1* and *GSTT1* genotypes were as follows: *GSTM1* (219 bp): 5’-GAACTCCCTGAAAAGCTAAAGC-3’ (forward) and 5’-GTTGGGCTCAAATATACGGTGG-3’ (reverse); *GSTT1* (459 bp): 5’-TTCCTTACTGGTCCTCACATCTC-3’ (forward) and 5’-TCACCGGATCATGGCCAGCA-3’ (reverse); and albumin (350 bp): 5’-GCCCTCTGCTAACAAGTCCTAC-3’ (forward) and 5’-GCCCTAAAAAGAAAATCGCCAATC-3’ (reverse). A total PCR reaction volume of 20 μL containing 25 units/mL Taq DNA polymerase, 200 μM dNTPs, 1.5 mM MgCl_2_, 0.25 μM of each primer, nuclease-free water, and 89 ng of genomic DNA was prepared. The desired genes were amplified under the following PCR conditions: 95°C (1 min), 60°C (1 min), 72°C (1 min) for 40 cycles, with a final extension step at 72°C for 10 min in a PTC-200 thermal cycler (MJ Research, Inc., Waltham, USA). All PCR products were separated by electrophoresis on a 1% agarose gel containing SYBR™ Safe DNA gel stain (Thermo Fisher Scientific, Waltham, USA).

*Statistical Analysis*

To determine the prevalence of *GSTM1* and *GSTT1* genotypes, genotype frequencies were computed, including deviations from the Hardy–Weinberg Equilibrium (HWE). The Pearson χ^2^ test was used to compare the differences in genotype frequencies between breast cancer cases and controls, while the relative risks of genotypes for breast cancer cases were determined by computing for the crude odds ratio (OR) with a 95% confidence interval (CI). *GSTM1 *and *GSTT1* genotypes were further correlated with other well-established risk factors for breast cancer.

## Results

**Figure 1:**
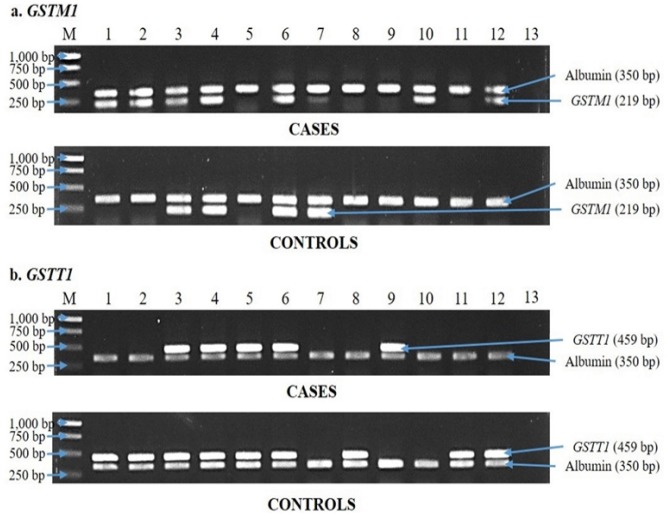
Representative Results for (a) *GSTM1* and (b) *GSTT1* Genotyping by PCR Method. DNA isolates from breast cancer cases and controls were subjected to duplex PCR using *GSTM1, GSTT1*, and albumin primers. (a) *GSTM1*. Cases: Lane M: marker; lanes 5, 7 to 9, and 11: *GSTM1* null; lanes 1 to 4, 6, 10, and 12: *GSTM1* positive; lane 13: negative control. Controls: lanes 1, 2, 5, and 8 to 12: *GSTM1* null; lanes 3, 4, 6, and 7: *GSTM1* positive. (b) *GSTT1*. Cases: Lane M: marker; lanes 1, 2, 7, 8, and 10 to 12: *GSTT1 *null; lanes 3 to 6 and 9: *GSTT1* positive; lane 13: negative control. Controls: lanes 7, 9, and 10: *GSTT1* null; lanes 1 to 6, 8, 11, and 12: *GSTT1* positive

This study performed *GSTM1* and *GSTT1* genotyping in 136 breast cancer cases and sex- and age-matched 136 clinically healthy controls by multiplex PCR. The presence of a 219 bp *GSTM1* (Figure 1a) or a 459 bp *GSTT1* (Figure 1b) PCR product, with reference to 350 bp albumin (positive internal control) indicates a “positive” genotype, while their absence indicates a “null” genotype. Since this study used conventional genotyping, it was not able to distinguish *GSTT1* and* GSTM1* positive genotypes as heterozygous or homozygous genotypes. 

**Table 1:** Comparison of Relative Risks of Breast Cancer Cases and Controls with Combinations of *GSTM1* Positive and Null Genotypes and Risk Factors

Characteristics	*GSTM1 *positive				*GSTM1* null			
	Cases n (%)	Controls n (%)	OR (95% CI)*	p-value†	Cases n (%)	Controls n (%)	OR (95% CI)*	p-value†
Alcohol Use								
Drinker	8 (13.8)	8 (18.6)	0.70 (0.24-0.04)	0.51	10 (12.8)	22 (23.7)	0.47 (0.21-1.08)	0.07
Non-drinker	50 (86.2)	35 (81.4)	1		68 (87.2)	71 (76.3)	1	
Tobacco Use								
Active								
Smoker	6 (10.3)	4 (9.3)	1.13 (0.30-4.26)	0.86	6 (7.7)	7 (7.5)	1.02 (0.33-3.18)	0.97
Non-smoker	52 (89.7)	39 (90.7)	1		72 (92.3)	86 (92.5)	1	
Passive								
Yes	29 (50.0)	18 (41.9)	1.39 (0.63-3.08)	0.42	49 (62.8)	37 (39.8)	**2.56 (1.38-4.75)**	**0.003**
No	29 (50.0)	25 (58.1)	1		29 (37.2)	56 (60.2)	1	
Family History of Cancer								
Immediate								
Yes	9 (15.5)	11 (25.6)	0.53 (0.20-1.43)	0.21	17 (21.8)	19 (20.4)	1.09 (0.52-2.27)	0.83
No	49 (84.5)	32 (74.4)	1		61 (78.2)	74 (79.6)	1	
Extended								
Yes	17 (29.3)	14 (32.6)	0.86 (0.37-2.01)	0.73	31 (39.7)	25 (26.9)	1.79 (0.94-3.42)	0.08
No	41 (70.7)	29 (67.4)	1		47 (60.3)	68 (73.1)	1	

*OR, crude odds ratio at 95% confidence interval; †Chi-squared test, two-sided p-value<0.05

**Table 2:** Comparison of Relative Risks of Breast Cancer Cases and Controls with Combinations of *GSTT1* Positive and Null Genotypes and Risk Factors

Characteristics	*GSTT1* positive				*GSTT1* null			
	Cases	Controls	OR (95% CI)*	p-value†	Cases	Controls	OR (95% CI)*	p-value†
	n (%)	n (%)			n (%)	n (%)		
Alcohol Use								
Drinker	8 (10.7)	18 (23.4)	0.39 (0.16-0.97)	0.04	10 (16.4)	12 (20.3)	0.77 (0.30-1.94)	0.58
Non-drinker	67 (89.3)	59 (76.6)	1		51 (83.6)	47 (79.7)	1	
Tobacco Use								
Active								
Smoker	10 (13.3)	9 (11.7)	1.16 (0.44-3.04)	0.76	2 (3.3)	2 (3.4)	0.97 (0.13-7.09)	0.97
Non-smoker	65 (86.7)	68 (88.3)	1		59 (96.7)	57 (96.6)	1	
Passive								
Yes	40 (53.3)	28 (36.4)	**2.00 (1.05-3.83)**	**0.04**	38 (62.3)	27 (45.8)	1.96 (0.95-4.06)	0.07
No	29 (46.7)	49 (63.6)	1		23 (37.7)	32 (54.2)	1	
Family History of Cancer								
Immediate								
Yes	16 (21.3)	15 (19.5)	1.12 (0.51-2.47)	0.78	11 (18.0)	15 (25.4)	0.65 (0.27-1.55)	0.33
No	59 (78.7)	62 (80.5)	1		50 (82.0)	44 (74.6)	1	
Extended								
Yes	27 (36.0)	24 (31.2)	1.24 (0.63-2.44)	0.53	21 (34.4)	16 (27.1)	1.41 (0.65-3.08)	0.39
No	48 (64.0)	53 (68.8)	1		40 (65.6)	43 (72.9)	1	

*OR, crude odds ratio at 95% confidence interval; †Chi-squared test, two-sided p-value<0.05

**Figure 2:**
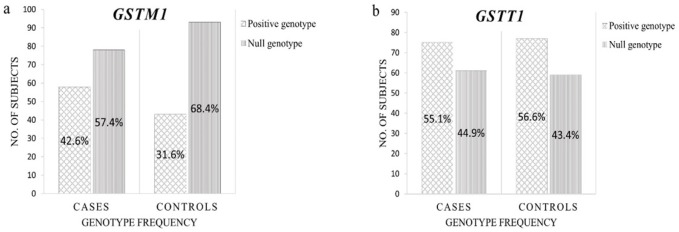
Comparison of *GSTM1* and *GSTT1* Genotype Frequencies. Frequencies were determined from the distribution of the positive and null genotypes of *GSTM1* and *GSTT1* to breast cancer cases and controls. (a) The frequencies of the *GSTM1* positive genotype for cases and controls were 42.6% (n=58) and 31.6% (n=43), respectively, while the frequencies of the *GSTM1 *null genotype for cases and control were 57.4% (n=78) and 68.4% (n=93), respectively. (b) The frequencies of the *GSTT1 *positive genotype for cases and controls were 55.1% (n=75) and 56.6% (n=77), respectively, while the frequencies of the *GSTT1* null genotype for cases and control were 44.9% (n=61) and 43.4% (n=59), respectively

There was no significant difference (p>0.05) in the frequency of *GSTM1* null genotype (Figure 2a) between cases (n=78; 57.4%) and controls (n=93; 68.4%). Similarly, frequency of *GSTT1* null genotype (Figure 2b) was not significantly different (p>0.05) between cases (n=61; 44.9%) and controls (n=59; 43.4%). The observed *GSTM1* and *GSTT1 *genotype frequencies did not significantly deviate from their expected frequencies, thus, they are consistent with the Hardy-Weinberg equilibrium. 

This study also determined the combined effect of the genotypic variants with lifestyle factors and family history of cancer. Only passive smoking was shown to be associated with increased risk of developing breast cancer. Among the cases carrying the *GSTM1* null genotype, the passive smokers had higher risk (OR=2.56; 95% CI=1.38-4.75) compared to active smokers (Table 1). A two-fold increased risk (OR=2.00; 95% CI=1.05-3.83) was also seen in *GSTT1* positive cases exposed to passive smoke (Table 2).

## Discussion

Breast cancer remains to be the leading cause of cancer among women worldwide, with over 2 million new cases resulting in more than 600,000 deaths each year. It is further estimated that by 2050, around 3.2 million women worldwide will be affected by breast cancer (Moore et al., 2008). In the Philippines, 14,955 newly diagnosed cases were reported in Metro Manila and Rizal Province between 2008 to 2012 (Bray et al., 2017). 

Several studies have shown the potential association of genetic polymorphisms with susceptibility to various types of cancer. Among the genes being extensively studied are *GSTM1* and *GSTT1*, which belong to the glutathione S-transferase (GST) supergene family. GSTs primarily catalyze the oxidation of glutathione, which subsequently detoxifies xenobiotics and carcinogens to make them easily excretable (Boyer, 1989). The *GSTM1* and* GSTT1* genes encode the GST μ (mu) and θ (theta) isoenzymes, *GSTM1* and *GSTT1*, respectively. The *GSTM1* isoenzyme is implicated in the cleansing of activated carcinogens such as hydroxylated metabolites of benzo[a]pyrene, epoxides, and polycyclic aromatic hydrocarbons (PAHs) (Ketterer et al., 1992), rendering these compounds more water-soluble for easy excretion outside the cell. The *GSTT1* isoenzyme is involved in the detoxification of numerous low molecular weight toxins such as ethylene oxides and butadienes, and haloalkanes including dichloromethanes (Guengerich et al., 1995; Hayes et al., 2005). 

It has been proven that homozygous deletions of the polymorphic *GSTM1* and *GSTT1* genes result in the loss of catalytic activity of the enzymes they encode correspondingly, leading to the decreased ability of the cells to detoxify various xenobiotic or genotoxic agents (Norppa, 2004). Exhaustion of glutathione to ~20-30% of the total glutathione level can weaken defense against xenobiotics, causing harm to several cellular processes (Reed, 1990). Hence, depletion of glutathione, which may be caused by the combined actions of all the GSTs, can expose the cells to damaging effects of oxidative stress and related mutagenic lesions. 

Results of this study show no significant difference in the frequencies of *GSTM1* and *GSTT1* null genotypes between Filipino breast cancer cases and clinically healthy controls. A slightly higher prevalence of the *GSTM1* null genotype in controls (68.4%) compared to the cases (57.4%) (Figure 2a) was seen in this study. Seemingly, the loss of the *GSTM1* gene might even offer a protective effect for the cell. This observation might be attributed to the interdependence among detoxifying and/or DNA-repair genes (Roodi et al., 2004). While studies have demonstrated that complete absence of *GSTT1* results in diminished detoxification capabilities (Pemble et al., 1994; Wiencke et al., 1995), this study shows lack of association between the *GSTT1* null genotype and the risk of developing breast cancer (Figure 2b).

Findings of this study are contrary to what have been seen among Indians (Kimi et al., 2016), Brazilians (Possuelo et al., 2013), Caucasians (Zheng et al., 2002), Dutch (van der Hel et al., 2004), and French (Lizard-Nacol et al., 1999). The lack of association of *GSTM1* null genotype with breast cancer has also been seen in Iranian (Saadat et al., 2001), Lebanese (Zgheib et al., 2013), Pakistani (Sohail et al., 2013), Taiwanese (Chang et al., 2006), Icelandic (Gudmundsdottir et al., 2001), Mexican (Rodriguez et al., 2014), and Caucasian and African-American (Van Emburgh et al., 2008) populations. The lack of association between *GSTT1* null genotype and breast cancer has been seen in Dutch (van der Hel et al., 2004), Lebanese (Zgheib et al., 2013), Pakistani (Sohail et al., 2013), Icelandic (Gudmundsdottir et al., 2001), Mexican (Rodriguez et al., 2014), Caucasian and African-American (Van Emburgh et al., 2008) and Iranian (Hashemi et al., 2012) populations. Association of *GSTT1* null genotype with breast cancer have been observed in studies from Northeast India (Kimi et al., 2016), Iowa (Zheng et al., 2002), Southern Taiwan (Chang et al., 2006), and China (Tang et al., 2015; Xiao et al., 2015; Song et al., 2016). 

Although this study shows lack of association of the *GSTM1* and *GSTT1* null genotypes with breast cancer, it should be noted that an interplay of genetic predisposition with environmental and lifestyle factors plays a role in increased susceptibility to breast cancer. Reproductive factors, which include nulliparity, family history of breast cancer, use of oral contraceptives, and age at menopause and menarche, are well-established risk factors for breast cancer. Lifestyle practices such as tobacco smoking, alcohol consumption, sedentary existence, and fatty diet are also among the predisposing factors (Ngelangel et al., 2009).

Passive smoking appears to increase the risk of developing breast cancer among Filipinos carrying the *GSTM1* null (Table 1) and *GSTT1* positive (Table 2) genotypes. Several compounds had been identified in secondhand tobacco smoke to be associated with cancer development (Margham et al., 2016). Our finding on the association of passive but not active smoking with risk of developing breast cancer may be attributed to the fact that the fume inhaled by passive smokers come from the unfiltered lighted end of the cigarette (sidestream smoke), hence, it contains more carcinogenic compounds than inhaled or mainstream smoke. Active smokers inhale filtered mainstream smoke that have reduced amount of tar and nicotine (Kapp, 2005). The minute size of nitrosamines and other carcinogens allow them to remain longer in the atmosphere. Vapor-phase constituents of passive smoke compared to particulate-phase constituents of mainstream smoke can also easily access the cells of the body and become readily absorbed into the blood and lymph systems (Li et al., 2015). Apparently, the *GSTT1* positive genotype combined with alcohol consumption showed a protective effect against the risk of breast cancer. This suggests the possibility that the *GSTT1* positive genotype is able to compensate the harmful effects of excessive alcohol consumption, or this particular gene might be interacting with other detoxifying genes that are encoding phase I enzymes or phase II enzymes apart from GSTs (Leelakumari et al., 2005).

In conclusion, no association was found between *GSTM1* and *GSTT1* genetic polymorphisms and the risk of developing breast cancer among Filipinos. However, this risk was modified when individual genotypes of *GSTM1* and *GSTT1* were combined with risk factors such as alcohol use and passive smoking. Nevertheless, it is recommended that the number of participants is increased to further validate the results of this study. It is further suggested to recruit participants from other parts of the country in order to accurately represent the entire Filipino population. 

## Declarations of Interest

The authors declare no conflict of interest.
